# Neuroimaging with SPECT-MRI: a myth or reality?

**DOI:** 10.3934/Neuroscience.2023004

**Published:** 2023-03-31

**Authors:** Arosh S. Perera Molligoda Arachchige

**Affiliations:** Department of Biomedical Sciences, Humanitas University, Milan, Italy

**Figure 1.**
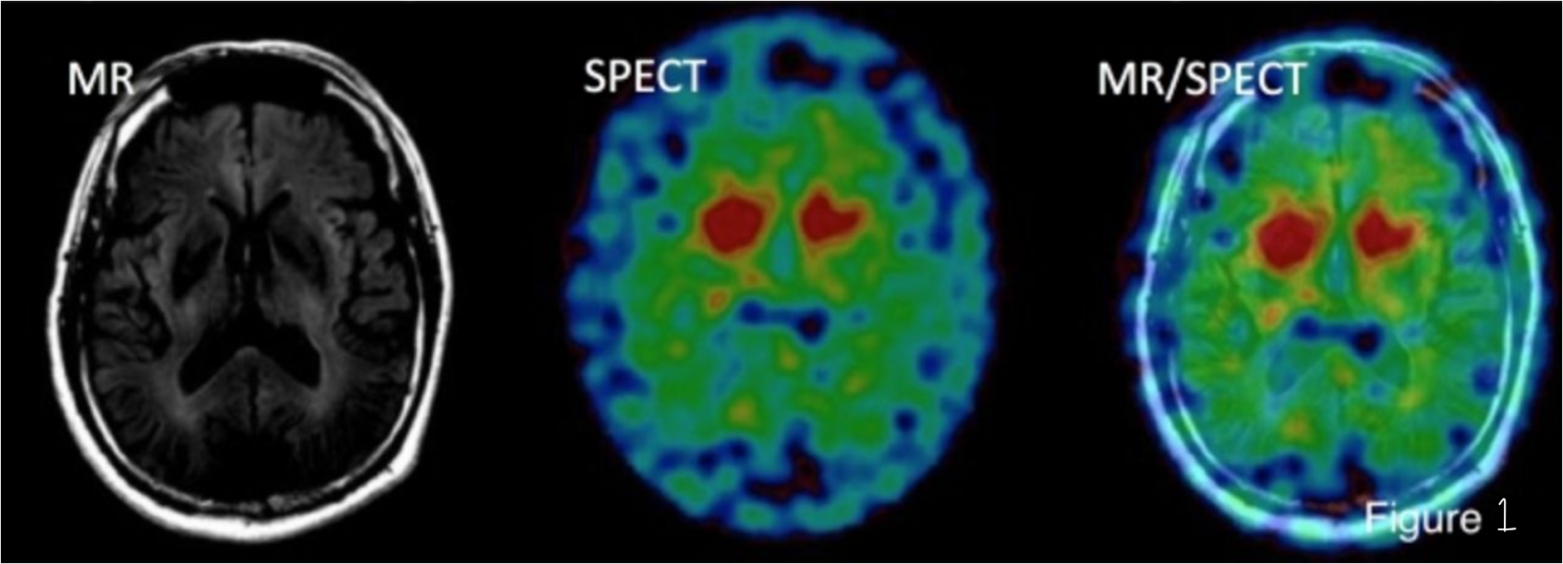
MR and SPECT images have been fused to create a MR/SPECT image. Adapted from Takeshi Hara et al. (2016) J Nucl Med. 57:1936.

Neuroimaging techniques are critical for the study of the human brain and the diagnosis and treatment of neurological disorders. Among these techniques, single-photon emission computed tomography (SPECT) and magnetic resonance imaging (MRI) are well-established methods for functional and structural imaging, respectively. The combination of these two techniques, known as SPECT-MR, has emerged as a promising modality for neuroimaging research and clinical practice [1] SPECT-MR combines the strengths of SPECT and MRI to provide a comprehensive view of the brain's functional and structural properties. SPECT relies on the injection of a radiotracer, which emits gamma rays that are detected by a gamma camera. This produces a 3D image of the distribution of the radiotracer in the brain, reflecting local blood flow or metabolism. MRI, on the other hand, uses strong magnetic fields and radio waves to produce high-resolution images of the brain's anatomy. In SPECT-MR, the SPECT data is co-registered and fused with the MRI data to create a single image that integrates functional and structural information. This is achieved by using specialized software that combines the SPECT and MRI data and allows for visualization of the functional changes in the context of the brain's structure. See Figure 1 [2].

A combined SPECT/MRI platform was first proposed in 2007 by Breton et al. who used a single pinhole SPECT system adjacent to a 0.1T magnet. The low MRI field strength made this solution suboptimal for use in routine preclinical research. However, since then systems combining SPECT and MRI have been introduced with both higher SPECT sensitivity and resolution and higher MRI field strengths [17]. To date, the SPECT/MRI system has been introduced to the market exclusively by MR Solutions (UK) allowing for sequential imaging of small animals at 9.4T, while simultaneous imaging systems are not yet commercially available [6]. One advantage of SPECT-MR is its ability to provide both functional and structural information of the brain in a single imaging session. This can reduce the need for multiple imaging sessions and increase patient comfort and convenience. Additionally, SPECT-MR has a higher spatial resolution than traditional SPECT imaging, which allows for more precise localization of functional changes within the brain [7]. SPECT is an attractive alternative to PET for preclinical studies due to its potential for better spatial resolution resulting from pinhole magnification. Additionally, SPECT allows for the use of multiple radionuclides that target different biomarkers and can use longer-lived and more accessible radionuclides. Operating costs for SPECT are also lower than for PET. Combining SPECT with MRI has further advantages, including providing high-contrast anatomical images that can be combined with SPECT acquisitions and using multiple pulse sequences to provide complementary functional measures, such as MR-spectroscopy. The availability of high-resolution anatomy from MRI, which has superior soft tissue contrast compared to CT (and lacks ionizing radiation), can also aid in SPECT quantification with correction for partial volume effects [15],[16].

SPECT-MR has many potential applications in neuroscience research and clinical practice. One major application is in the diagnosis and management of neurological and psychiatric disorders. SPECT-MR can be used to identify patterns of brain activity associated with different disorders, such as Alzheimer's disease, epilepsy, and depression. This can lead to more accurate diagnoses and more effective treatment strategies [3],[4]. SPECT-MR can be used to identify the epileptogenic focus in patients with epilepsy. The process of co-registering ictal SPECT with MRI, similar to PET, can enhance its precision and ability to exhibit more accurate anatomical localization. This innovative method is known as subtraction ictal SPECT co-registered with MRI (SISCOM) and has been linked to favorable postoperative outcomes and eradication of seizures when used to identify the epileptogenic focus. Moreover, SPECT co-registered with MRI is beneficial in pinpointing the precise location for the placement of intracranial EEG leads during invasive recording, thereby minimizing potential complications. It is also capable of detecting the ictal focus with high sensitivity in cases of extratemporal lobe epilepsy [5]. SPECT-MR can be used to study the regional cerebral blood flow in patients with Alzheimer's disease. By combining SPECT with MR, it is possible to identify the specific regions of the brain that show reduced blood flow and metabolism, allowing for targeted treatment [8]. SPECT-MR can be used to study the dopaminergic system in patients with Parkinson's disease. The SPECT component can provide functional information about the dopaminergic system, while the MR component can provide high-resolution anatomical details. Also, it has been shown that advanced MRI techniques as diffusion-weighted imaging, diffusion-tensor imaging, MR-spectroscopy, magntization-transfer imaging, and magnetic resonance imaging-based volumetry are more delicate in separating PD from atypical parkinsonian syndromes. By combining these two imaging modalities, clinicians can precisely locate the area of abnormal dopaminergic activity, which is critical in the diagnosis and management of Parkinson's disease [9],[10]. SPECT-MR can be used to study the metabolism and blood flow in brain tumors. The SPECT component can provide functional information about the tumor's blood flow and metabolism including receptor binding, uptake and metabolism of exogenous compounds, heamodynamics, etc. while the MR component can provide high-resolution anatomical details. By combining these two imaging modalities, clinicians can accurately locate the tumor and assess its growth and response to treatment [11]. Another application of SPECT-MR is in the study of brain function and cognition. SPECT-MR can be used to map changes in brain activity associated with specific tasks, such as language processing, memory encoding, and emotion regulation. This can help researchers understand how the brain works and how different regions of the brain interact with each other [12]. SPECT-MR also has potential applications in the study of drug development and pharmacology. By mapping changes in brain activity associated with different drugs, SPECT-MR can help researchers identify new therapeutic targets and evaluate the efficacy of different treatments [13].

However, there are also some limitations to SPECT-MR. SPECT-MR is a relatively new imaging technique, and as such, it is not widely available in all hospitals and medical centers. This is also related to the cost and complexity of the technique, which may make it less accessible to some researchers and clinicians. One limitation is its relatively low temporal resolution since SPECT is dependent on the haemodynamic response and sufficient detection of photon emissions, which limits its ability to capture rapid changes in brain activity. In addition, there are technical challenges: SPECT-MR requires specialized expertise and equipment. The complexity of the imaging process and the need for advanced data analysis can lead to technical challenges that require highly skilled personnel [14].

SPECT-MR is a promising imaging modality that has the potential to advance our understanding of the brain and its disorders. While there are still some technical and practical limitations to be overcome, ongoing research and development in this area hold great promise for the future of neuroscience research and clinical practice. The integration of functional and structural imaging with SPECT-MR represents a significant step forward in neuroimaging and has the potential to improve our understanding of brain function and lead to better diagnoses and treatments for neurological and psychiatric disorders [15].
